# Network Analysis of Small Ruminant Movements in Uganda: Implications for Control of Transboundary Animal Diseases

**DOI:** 10.1155/tbed/7474495

**Published:** 2025-05-14

**Authors:** Joseph Nkamwesiga, Karla Rascón-García, Paul Lumu, Henry Kiara, Andres Perez, Dennis Muhanguzi, Kristina Roesel

**Affiliations:** ^1^Department of Veterinary Medicine, Institute of Virology, Freie Universität Berlin, Berlin, Germany; ^2^Health Program, International Livestock Research Institute, Nairobi, Kenya; ^3^Center for Animal Disease Modeling and Surveillance, Department of Medicine and Epidemiology, School of Veterinary Medicine, University of California, Davis, California, USA; ^4^Department of Animal Health, Ministry of Agriculture, Animal Industry and Fisheries, Entebbe, Uganda; ^5^Department of Veterinary Population Medicine, College of Veterinary Medicine, University of Minnesota, St. Paul, Minnesota, USA; ^6^Department of Biomedical Laboratory Technology and Molecular Biology, College of Veterinary Medicine, Animal Resources and Biosecurity, Makerere University, Kampala, Uganda; ^7^Department of Animal Breeding and Husbandry in the Tropics and Subtropics, University of Hohenheim, Stuttgart, Germany

**Keywords:** goats and sheep, livestock mobility, targeted control interventions

## Abstract

Domestic animals are moved for reasons that are mutually beneficial to the animal and the farmer. Some examples include the need for fresh grazing grounds and watering points, or the need to access livestock markets for income to sustain farmers' livelihoods. However, livestock mobility is a key risk factor for the transmission of transboundary animal diseases. Contact tracing of individual animals and flocks is very challenging, especially in most low-income countries, due to a lack of efficient livestock traceability systems. Despite these challenges, low-income countries, such as Uganda, issue paper-based animal movement permits (AMPs) to ensure only clinically healthy animals are moved following a physical inspection. In this study, we used national approximately 9 years of (2012–2020) small ruminant movement data obtained from archived AMPs in Uganda to describe small ruminant movement networks. The movement networks were described using social network analysis (SNA) approaches implemented in R software to identify and visualize relationships between individual and groups districts in Uganda. Lira, Kaberamaido, Nabilatuk, Mbarara, Kiruhura, Kampala, and Wakiso were identified as districts with the highest degree (in and out-degree) and betweenness among other centrality measures. Our results suggest these districts could be the most important bridges connecting the various regions of the country. Tailoring control interventions to such districts with high incoming and high outgoing shipments, or bridges, would accelerate the nation's ability to timely detect outbreaks, prevent or mitigate further spread, and contain diseases in their original foci, respectively. We also identified areas for active surveillance, vaccination, quarantine, and biosecurity measures-staging depending on prevailing circumstances. These findings will be used to guide the national small ruminant infectious diseases control strategies and subsequently contribute to national and global initiatives, such as the 2030 Peste des petits ruminants (PPR) eradication program.

## 1. Introduction

Livestock mobility is one of the major epidemiological risk factors for the transmission and spread of infectious diseases of veterinary and public health concern [1 ]. Livestock are often moved from place to place for purposes of trade, social functions, drought copying strategy especially among pastoral communities, and flock breed improvement within and outside international boundaries [2]. In Uganda, most animals are moved between districts from within the country and, to a lesser extent, across the frontiers through legal and illicit trade, especially at porous international borders [3, 4].

Seasonal variations strongly influence livestock movements across districts in Uganda, as well as, across international borders. During the dry season, animals from semiarid areas are moved in search for fresh pastures and water [5]. The majority of livestock in Uganda are moved from rural to urban and peri-urban areas for slaughter, owing to the increased demand for livestock and livestock products [6]. Additionally, livestock are borrowed or gifted between households for social functions and social security; sometimes, these transactions occur across international borders, especially in communities that occupy areas that lie across international borders [4, 7, 8].

In Uganda, there are more small ruminants (21.8 million total estimated; 17.4 million goats and 4.4 million sheep) than there are cattle (14.5 million). This is due to small ruminants' ability to multiply quickly, making them easier to convert to cash for farmers to take care of urgent basic needs like children's school fees, food, healthcare, and other livelihood needs [9]. However, the high burden of livestock diseases in Uganda directly affects livestock productivity and the general livelihoods of the livestock keeping communities [10, 11]. Furthermore, the risk of zoonotic disease transmission is also very high because of the close contact that occurs between livestock and their caregivers during grazing, watering, milking, and transportation events. These activities, coupled with poor biosecurity measures, especially in districts with high levels of livestock mobility, further aggravate the risk of zoonotic transmissions [12–14].

Even though livestock movement positively contributes to the livelihoods of farmers and all those involved in the livestock value chain, its contribution to transmission and spread of infectious livestock diseases (within and beyond national borders) cannot be ignored. For example, the first confirmed outbreak of Peste des petits ruminants (PPR) in Burundi during the year 2018 coincided with movement of an exotic breed of goats from western Uganda to Burundi through Tanzania in a project aimed at improving goat production in Burundi [15, 16]. Movement of infected animals between premises, such as farms and livestock markets, was also reported as one of the most important factors that contributed to the first outbreak of foot and mouth disease in Uruguay [17]. Infectious diseases, such as, PPR heavily rely on the close contact between susceptible and infectious small ruminants for their successful transmission. Unrestricted animal movements are a potential threat to the introduction and reintroduction of such diseases across geographical areas in the shortest time possible [1, 18].

Animal mobility data, if well collected through accurate animal traceability systems, can be an integral tool in the management and control of livestock infectious diseases. However, in most African countries, including Uganda, no such systems exist. Instead, paper-based animal movement permits (AMPs) are issued for animals relocating for commercial purposes but less so for local animal movements purposes, such as grazing and watering [19]. Animals are trekked for relatively short distances, such as to nearby markets within districts, or moved for longer distances on trucks for different purposes including trade, breeding, and slaughter. In such resource-constrained settings, AMPs are often issued to generally ensure that (1) only healthy animals are moved and (2) to collect government taxes as a modest amount of money is levied on every individual animal moved [2]. The AMP data are often incomplete because of either noncompliance, non-vigilance of attending veterinarians, or due to poor archiving methods, which result in unprecedented gaps in the data that are difficult to account for [19]. Missing data in these AMPs with long periods of no animal movements recorded makes it difficult to determine whether these are true observations (i.e., no animals were moved during these periods), or whether movements were simply not recorded [19]. Nonetheless, data from AMPs have previously been incorporated into the social network analytic workflows to identify critical areas for surveillance and targeting of control interventions [5, 19].

According to Guinat et al. [20], the relationship between “actors” or “nodes” and how they are connected to one another (or “edges”) may help forecast the spread of diseases and, at its core, should offer information about the scale of prospective epidemics. This information may also be used to enhance surveillance and control tactics. Social network analysis (SNA) methodologies allow for the identification of important (“central”) nodes (e.g., epidemiological units, such as individuals, farms, etc.). In the context of this study, we describe the relationship between nodes through their “edges” (i.e., observed animal shipments, frequency of shipments, number of animals moved per shipment, etc.) in an animal movement network of small ruminants within Uganda. Through SNA, we describe important players' influence and “centrality” in the larger movement network by examining the level of “connectedness” each node is suggested to be by use of standard SNA metrics, as previously described [21–23].

Previous studies in Uganda used recent (2019–2021) AMPs that may have had interruptions caused by the COVID-19 lockdown periods and, consequently, only considered a few years to describe animal movement networks [6, 24]. In this study, we set out to conduct network analyses to describe small ruminant (goats and sheep) movement networks over the approximate 9-year period (2012–2020) to understand the flow of small ruminants across the four different regions of Uganda (Central, Northern, Eastern, and Western) and their respective subregions. This work, in turn, identifies the critically important districts in each region (or subregion) which should be targeted for small ruminant surveillance and other relevant control interventions to minimize or even block transmission of transboundary animal diseases for example PPR.

## 2. Materials and Methods

### 2.1. Data Source

**Figure 1:**
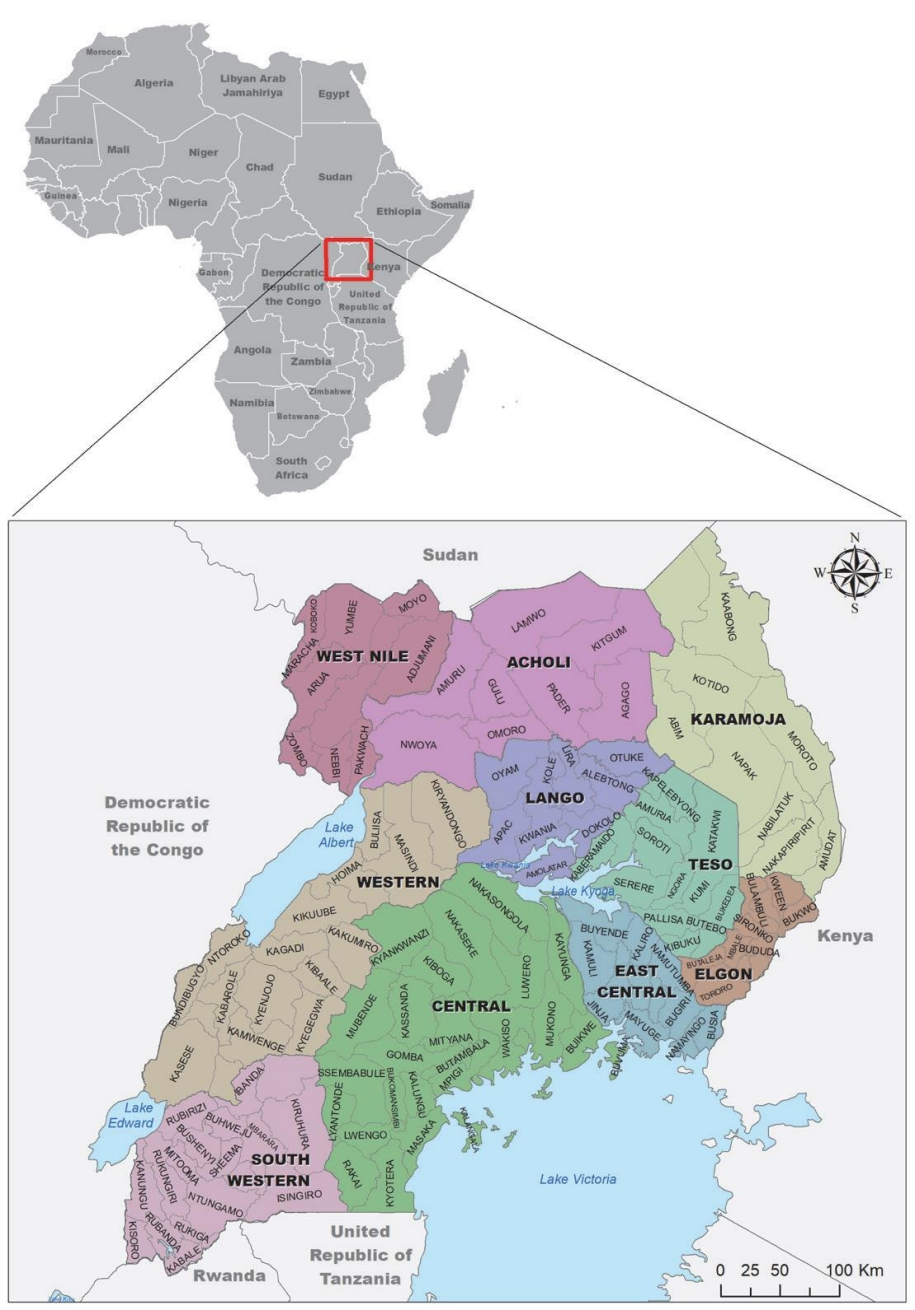
Map of Uganda showing the different administrative districts and subregions. The map was adapted from a study [25].

This study was primarily based on archived AMPs for the past 9 years (2012–2020) retrieved from Uganda's Ministry of Agriculture Animal Industry and Fisheries (MAAIF). These permits were digitized in Microsoft Excel to capture key movement attributes, such as the district of origin and destination, date on which animals were moved, number and species of livestock moved, as well as mode of movement or transportation, and purpose for the recorded movement. By the time of the study data endpoint (2020), Uganda had 137 districts distributed across 10 subregions with related within-region socioeconomic and demographic characteristics (Figure 1).

### 2.2. Data Analysis

In the following network analysis, the individual districts are referred to as “nodes” where connections between districts (i.e., “edges”) represent the event of an animal shipment between two districts. Here, a directed network graph illustrates the interconnectedness between districts by means of animal movements from districts of origin to associated destinations. In contrast, an undirected network is a graph where there is no explicit trajectory or direction in the movement; edges could be bidirectional and are often represented with no arrows.

The relative importance of districts or groups of districts was described using social network centrality measures. Network centrality measures are a critical tool for quantitatively describing the relative importance of either, (1) a given node or individual to other nodes (i.e., node-level centrality) or (2) group of nodes (graph-level centrality) in a network. Different centrality metrics have been developed and can be implemented depending on whether the graph is directed or not. However, it is important to note that a node can be “highly central” by one measure and yet have very low centrality by another measure or definition. For instance, though *degree* centrality (see Table 1) gives a glimpse of how many unique neighbors a node has in an observed network, it does not necessarily comprehensively reveal how “important” that node is to other nodes, or the whole network. The interpretation of centrality measures strongly relies on the understanding and context of the analysis at hand. Some of the terminologies this study used to describe the small ruminant movement network are summarized in Table 1.

An analysis of variance (anova) was used to compare the different measures of centrality by subregion. Respective model residuals were tested for homogeneity of variance normality using the Levene and Shapiro tests, respectively. Finally, the Kruskal–Wallis test with Bonferroni correction was used to compare the metrics that were not normally distributed [31]. The quantity of small ruminants moved, and the frequency of movement transactions were summarized and visualized using *ggplot2* function in R software version 4.4.1 [32].

### 2.3. Modeling Approach

Two tables were prepared from the digitized AMP spreadsheets using the *dplyr* package from the *tidyverse* collection of packages [33]. One table (i.e., the *nodelist*) contained information on the unique districts that appeared as either an origin or destination district along with district-level attributes, such as region, subregion, and the estimated small ruminant population. The second table (i.e., the *edgelist*) contained details specific to the event or date on which small ruminants were moved between two districts in Uganda. The *edgelist* described the number of small ruminants moved, frequency of small ruminant movements, as well as the purpose and mode of transportation. All movement records were aggregated and summarized to describe monthly origin-destination transactions.

A static network was constructed from the prepared node- and edge-lists using the R software package network [34]. Each district's earliest onset and latest terminus time (i.e., the earliest and latest month each district was available to move or receive animals) was obtained from observed AMP movement dates. A dynamic network was then constructed with discrete monthly time-steps using the R package *networkDynamic* [35].

## 3. Network Visualizations and Centrality Measures

Static networks were visualized using the *igraph* package [36]. The *intergraph* package [37] was used to convert the network objects into *igraph* objects before computing node-level metrics. Node-level metrics, such as degree, indegree, outdegree, closeness, betweenness centrality, *eignenvector* centrality, authoritative score, and PageRank (Table 1) were computed using their corresponding functions implemented in the *igraph* package. Node-level attributes of the dynamic network, such as backward and forward reachability among other time series metrics were computed using the *tSnaStats* function from the R software package *tsna* [38]. Plots were generated using igraph while data were wrangled using *tidyverse* and visualized using *ggplot2* packages [33].

## 4. Results

**Figure 2:**
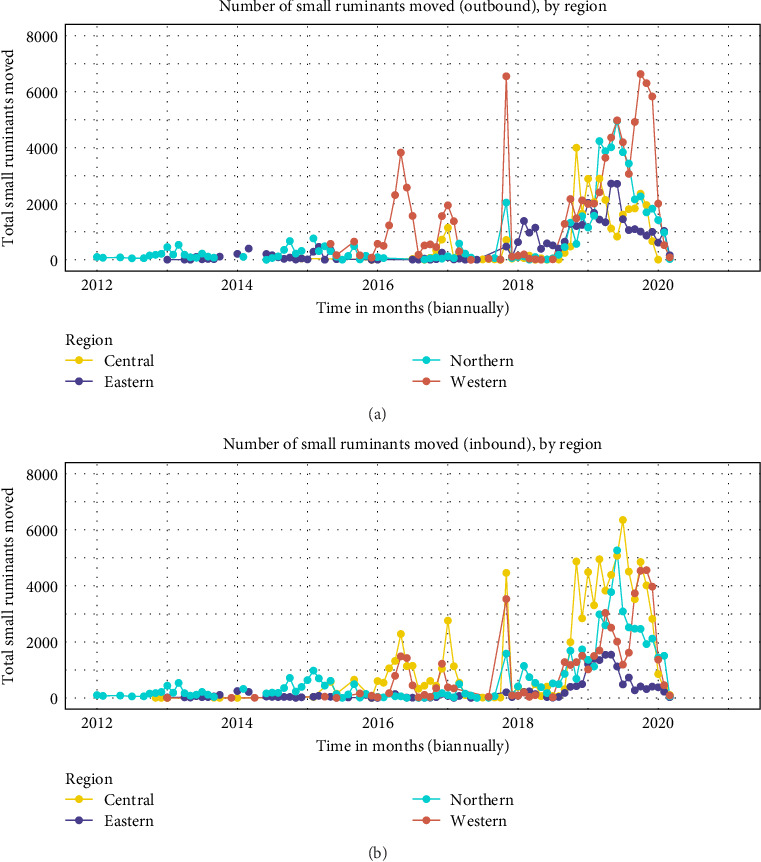
The total number of small ruminants moved from all 127 districts for a period of 95 months. Panel (a) shows the quantity of monthly outbound small ruminant moved between districts across the four regions of Uganda. Panel (b) shows the total number of inbound small ruminants. The movements were aggregated by a triplicate-variable containing origin—destination—month temporal windows and plotted on a biannual scale.

The retrieved AMPs contained records from January 18, 2012 to March 12, 2020. The AMP records spanned 95 monthly-time steps across 94% (127/135) of all districts in Uganda with 2642 unique transactions accounting for more than 200,000 small ruminants moved across Uganda. The total number of small ruminants moved (both in and outbound) per month generally increased over time from 2012 to 2020 across the different regions of Uganda. More specifically, a gradual increase in small ruminant movements were observed between January 2012 through January 2016 before a subsequent sharp rise around March 2016. The total number of animals moved continued to increase, resulting in a second sharp rise in animal movements around December 2017. The frequency of small ruminant movements followed a general increasing trend over time (Figure 2).

The highest volume of animals was consistently moved from 2018 to 2020. The Western region demonstrated the highest number of small ruminant movements with three temporal spikes attributed to this region across the study period. About 4000 small ruminants were moved from the western region around June 2016, whereas over 6800 small ruminants were moved from the western region around December 2017 and June 2020 respectively (Figure 2a). Generally, districts from the central region of Uganda dominated the receiving of small ruminants from the rest of the regions in Uganda. Mid 2016, 2017, 2018, and 2020 were the years in which the central region received the highest number of small ruminants. In terms of receiving animals, the central region was followed by the western and northern regions (Figure 2b).

**Figure 3:**
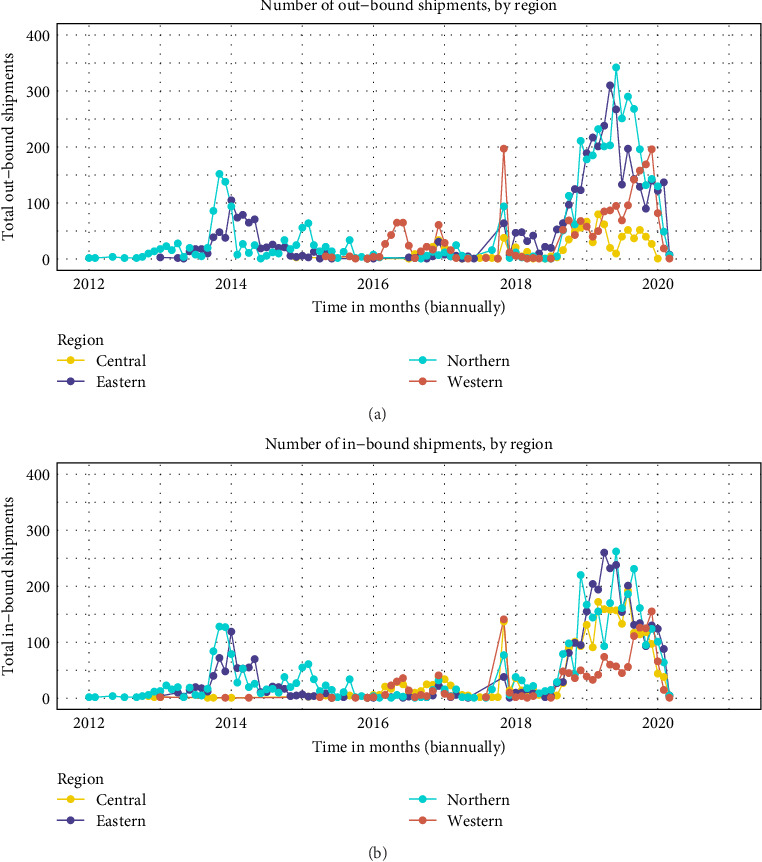
The total frequency of small ruminant shipments for all 127 districts for a period of 95 months. Panel (a) shows the frequency of monthly outbound small ruminant movements between districts across the four regions of Uganda. Panel (b) shows the total frequency of inbound small ruminant shipments. The movements were aggregated by a triplicate-variable containing origin—destination—month temporal windows and plotted on a biannual scale.

The number of outbound shipments within and between regions changed drastically over time with the initial shipments between the northern and eastern region albeit on a small scale around the year 2014. Between 2012 and 2015, outbound small ruminant movements occurred exclusively between northern and eastern regions before movements were dominated by the western region until last quarter of 2017, with 200 outbound movements per month being the highest in 2018. The highest frequency of outbound small ruminant movements (over 350) was observed in the northern region of Uganda, around 2019 (Figure 3a).

Similar trends were observed in the frequency of inbound small ruminant movements across the study period. Three distinct peaks of high frequency of inbound monthly small ruminant movements of around 120, 150, and over 200 were observed in 2014, 2018, and 2019 respectively. Frequency of inbound small ruminant movements was generally dominated by the eastern and northern regions of Uganda with the western and central region dominating only around the last quarter of 2017 (Figure 3b).

Small ruminants, and indeed other livestock, are relocated for purposes including slaughter, breeding, social functions (e.g., dowries), or research purposes, among others. Different means of transport for example trekking (movements by hoof), truck, boat, motorcycle, bicycle, and/or ferry were also observed depending on the convenience, distance and number of animals involved. The two most common reasons for moving small ruminants were for slaughter and breeding, which accounted for 73.8% (8588/11,631) and 26.1% (3037/11,631) of all small ruminant movement transactions, respectively. Social functions, like dowry, and research purposes contributed the least to the purpose of small ruminants' movement. Movements by trucks and trekking dominated the modes of transportation accounting for 72.7% (8457/11,631) and 20.6% (2392/11,631) of all transactions, respectively (Table 2).

**Figure 4:**
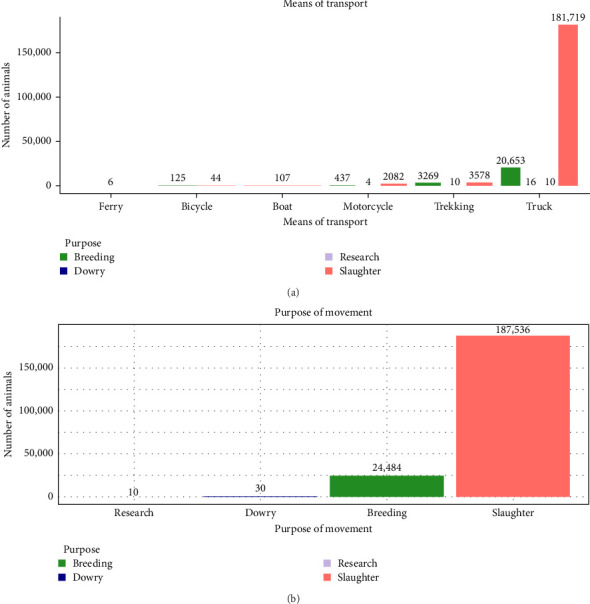
A summary of mode of transportation and purpose of small ruminant movement over time. Panel (a) shows a summary of the different *means of transport* available for movement of small ruminants in Uganda whereas panel (b) shows the summary of all the purposes motivating these movements. The numbers on top of each bar indicate the total number of small ruminants moved.

During the period 2012–2020, more than 200,000 small ruminants were moved across Uganda districts. Over 90% of the animals were moved in trucks, whereas the rest were either trekked or carried on motorcycles (Figure 4a). Similarly, over 90% (>150,000 animals) of all small ruminants were destined for slaughter while the rest were moved for other purposes including breeding, dowry, and research (Figure 4b).

Network-level metrics were computed to evaluate the importance of individual districts or groups of districts in the small ruminant movement network. The network size was 127 districts with a diameter of nine districts and an edge density of 0.165 over the entire study period. We observed a moderate to high tendency for districts to ship more animals between districts from the *same* region than outside the region (nominal assortativity: 0.495).

**Figure 5:**
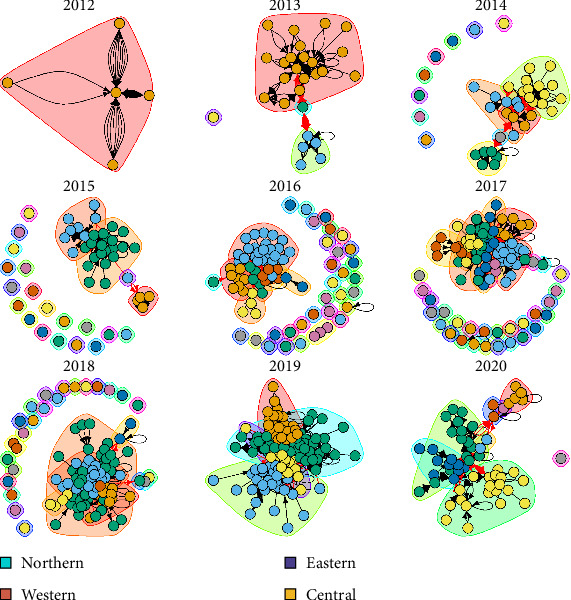
Small ruminant network communities over time (2012–2020). The dots represent individual districts, and their colors indicate the region to which the districts belong.

The monthly small ruminant movements between districts gradually increased from time to time. In the first year 2012, the small ruminant movement network was dominated by only districts from northern Uganda. Small ruminant movements to other regions were observed by the second year (2013) with western and northern regions dominating receipt of animals from within and between regions. The network was generally highly fragmented in the first few years with no strong tendencies of consistent partnering between districts. By the third year (2014), AMP records showed that the central region of Uganda joined the network and dominated the interactions as movement networks continued to grow as time went on and by 2020, small ruminants had been shared across all regions in Uganda. The communities formed across temporal cross-sections of the dynamic network of the small ruminant movement network from the earliest onset (first month) to the end of the study (last month) (Figure 5).

**Figure 6:**
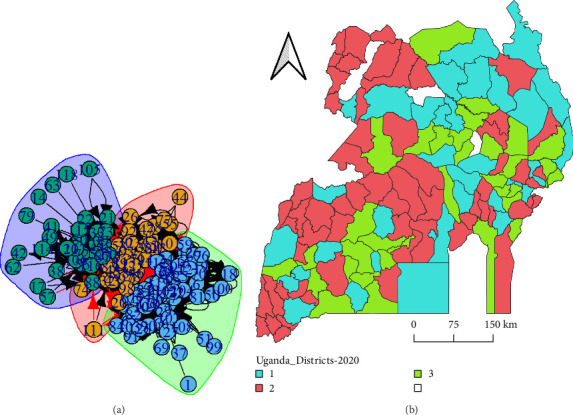
Community formation for the entire study period (2012–2020) (a). The different districts were colored by the community they belonged to (b). The map was drawn using open-access datasets from Uganda Burau of Statistics and open source QGIS software. The white space signifies the districts that were not associtaed with any of the 3 communities identied.

Different districts in Uganda formed three communities over the course of the aggregated 95-month time window (study period). Initially, the network was very fragmented over time (Figure 5); however, by the end of the study period, districts appeared to gravitate towards the formation of three tightly knit communities in which movement transactions occurred (Figure 6a). The communities consisted of districts that were generally spatially close to each other with a wide connection to spatially distant neighbors (Figure 6b).

**Figure 7:**
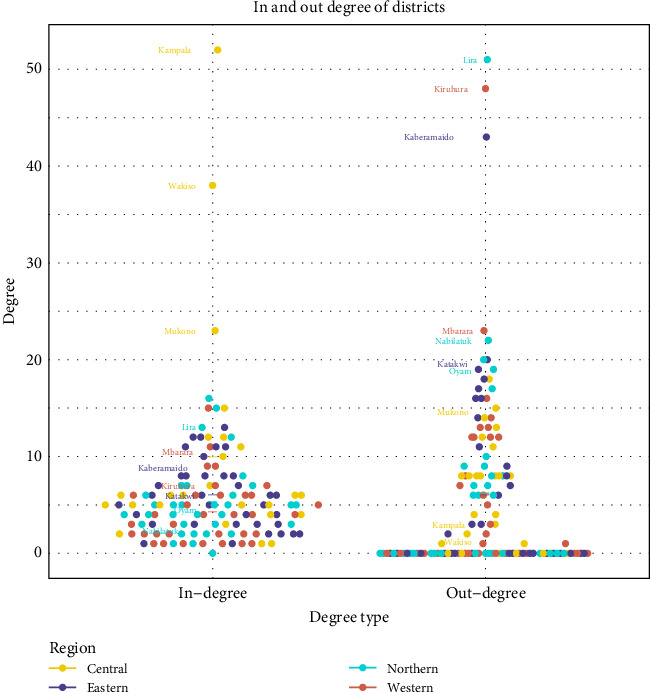
The dot plots show the in- and out-degree of different districts across regions of Uganda. The in-degree and out-degrees ranged from 0 to 52. The color of the dots corresponds to the region in which a district is from. The cross bar along the central axis of each dot plot represents the average degrees for each region.

Generally, districts participated more in receiving than sending out animals as revealed by a higher average in-degree than average out-degree. The mean in-degree for the entire network was about 42, suggesting that, on average, each district received animals from 42 unique districts. Districts from the central region had the highest average in-degree (in-degree: 10) followed by the eastern region with an average in-degree of 6, whereas the northern and western regions had the least in-degree averages of 5.3 and 4.3, respectively, suggesting lower levels of engagement. All districts with the highest tendency to receive animals (i.e., highest in-degree) were from the central region, including the highly populated Kampala, Wakiso and Mukono districts (Figure 7).

The average out-degree across all districts, on the other hand, was 6. The biggest sources of small ruminants to other districts (as defined by their out-degree) were Lira, Kaberamaido, Kiruhura, and Mukono for the northern, eastern, western, and central regions, respectively (Figure 7). The dominant small ruminant destinations in Uganda were the central (Kampala and Wakiso districts) and southwestern subregions (Mbarara and Kiruhura districts) whereas the eastern and northern regions were more of origins of small ruminants to other parts of the country (Figure 7).

**Figure 8:**
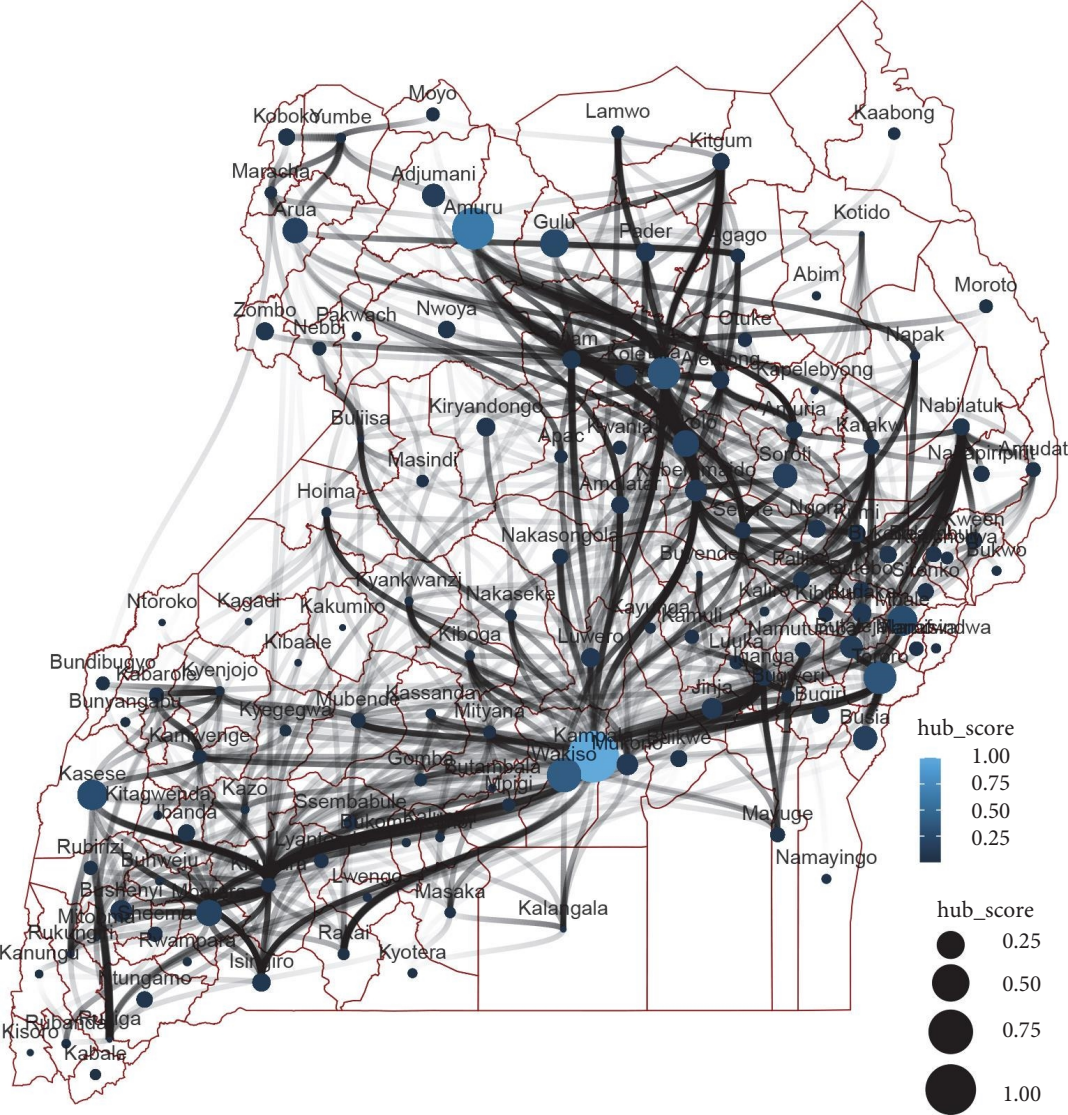
Static network for small ruminant movements in 127 districts of Uganda. Here, the size and color of the node is proportionally scaled to represent hub score centrality. The connections between districts are weighted based on the frequency of livestock movement between them. The map was generated in R version 4.4.2 using open-source shapefiles <https://www.ubos.org/data-portals-2/>.

Interestingly, Kampala and Wakiso districts in central Uganda had the highest hub scores of 100% and 63% respectively. Other Ugandan districts that constitute major towns including those that lie at international borders, such as Amuru (Acholi subregion), Gulu (Acholi subregion), Kasese (western subregion), Lira (Lango subregion), Mbale (Elgon subregion), and Tororo (Elgon subregion) were among the districts with hub scores of more than 30% (Figure 8).

A number of districts including Lira, Kiruhura, Tororo, and Kaberamaido had high authority score values (Supporting Information Figure S1a). Lira, Mukono, Mbarara, and Kiruhura districts were the identified as distrcits with the highest betweenness centrality scores ranging between 0.6 and 0.8 (Supporting Information Figure S1b). Kampala, Wakiso, Kiruhura, and Lira districts had the highest closeness centrality values as compared to other districts in Uganda (Supporting Information Figure S1c). Additionally, Lira and Alebtong districts from the Lango subregion in northern Uganda were among the most influential districts by eigenvector centrality scores of 1.0, 0.99, and 0.95 respectively (Supporting Information Figure S1d).

**Figure 9:**
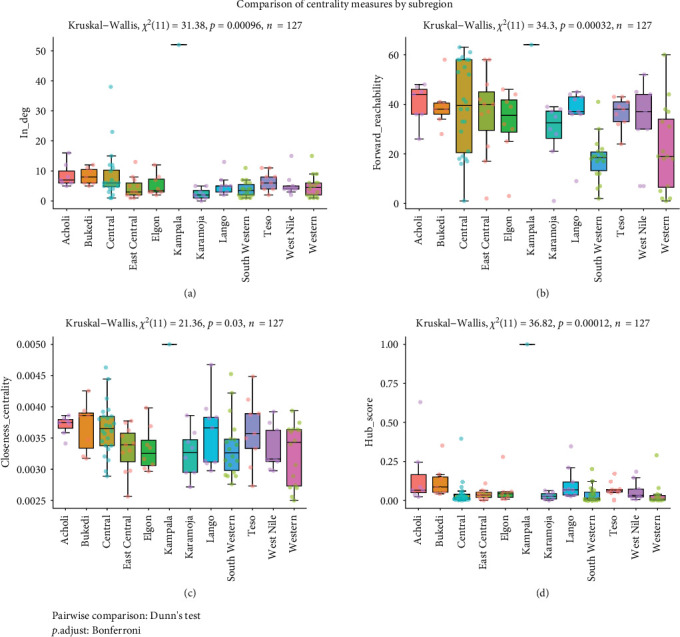
Box plots show the pairwise comparison of statistically significant measures by subregion. In-degree (a), forward reachability (b), Closeness centrality (c), and Hub score (d). The dots represent individual districts whereas dot colors represent the subregions from which a district is drawn.

Generally, districts received more animal shipments than they shipped out animals as the in-degree (Figure 9a) and backward reachability scores were higher than the downstream reachability across the study period (Figure 9b).

In-degree, forward reachability, closeness centrality and hub score values varied significantly (*p* < 0.05) across subregions of Uganda. Districts from central Uganda, such as Kampala, Wakiso, and Mukono, and those that lie at international borders, such as Amuru, Kasese, Mbale, and Tororo received disproportionately more small ruminants than other districts in Uganda (Figure 9a). Small ruminants from central and western Uganda districts reached more districts in Uganda than any other subregion. Closeness centrality varied significantly across Ugandan subregion (Figure 9b). Closeness centrality measures an individual node's average farness (inverse distance) to all other nodes in the network such that the higher the closeness values, the closest the node is to every other node. Closer nodes distribute information more rapidly and more efficiently. Districts from different regions had a statistically significant closeness centrality measure (Figure 9c). On average, districts from central Uganda including Kampala were closer to all other districts than those from the other regions and acted as the most important link for animal movements to trickle into other regions (Figure 9c). Hub score values significantly differed across subregions. On average, Kampala was the most significant hub for receiving small ruminants from equally important locations in Uganda. This was followed by Acholi subregion whereas the least hub score values were observed in the Karamoja subregion (Figure 9d).

**Figure 10:**
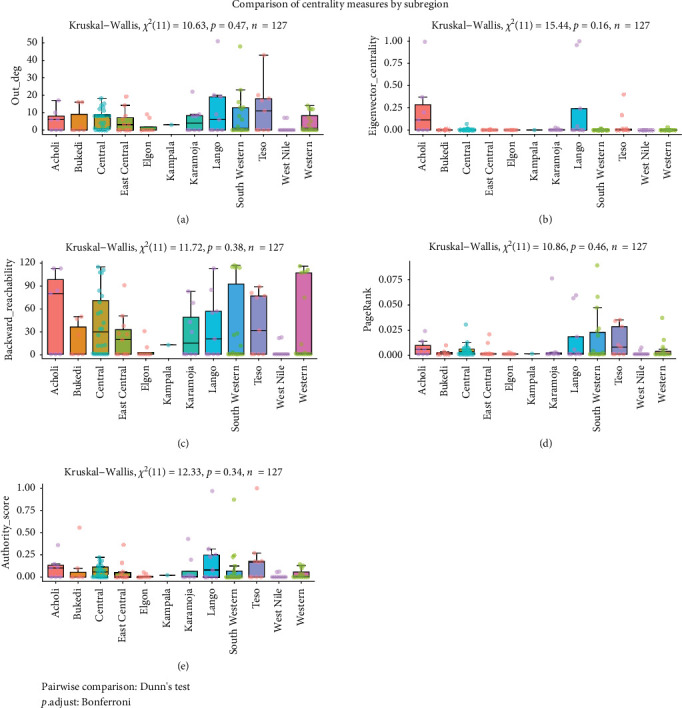
Box plots show the pairwise comparison of centrality measures by subregion- that were not statistically significant. out-degree (a), eigenvector centrality (b), backward reachability (c), pagerank (d),. and authority score (e). The dots represent individual districts whereas dot colors represent the regions from which a district is drawn.

Five measures including out-degree, authority score, betweenness, eigenvector centrality, backward reachability, were also compared across subregions, and were not statistically significant. However, some of the metrics revealed individual districts with extreme values that are worth noting (Figure 10).

## 5. Discussion

This study set out to identify the districts with the highest connectivity in the small ruminant movement network for the period 2012–2020 using the SNA approach. The available livestock mobility literature in Uganda is based on Animal Movement Permit (AMPs) data from only 2019 [6] and shortly after between 2019 and 2021 [24]. Both studies acknowledge a potential limitation that was posed by the COVID-19 related lockdown periods that negatively impacted livestock trade that saw a sharp decline in demand for livestock and livestock products leading to majority of key players diversifying to other economic activities [39]. This study builds on already existing literature to expound more on small ruminant movement networks across all districts of Uganda for a longer period (2012–2020).

Influential districts in the animal movement network can be a basis for targeted control interventions, such as vaccination, quarantine, and biosecurity measures. The most important districts and activities in the small ruminant as well as all livestock movements in Uganda were those with the highest levels of connectivity through network centralization measures, namely degree, betweenness, closeness, hub score among others. On average, for every centrality measure tested in this study, there were notable outlier districts. Such outliers have previously been described as super-spreaders of infections as these are the individuals onto which the entire network is anchored [40]. There were more districts with outgoing animal shipments than incoming shipments, an observation similar to what has been reported by another study on cattle movements in Uganda [24].

The districts with exceptionally higher levels of incoming animals imply that they are at increased risk of receiving infected animals and thus could be very important for surveillance activities depending on the purpose of movement [23]. For example, most small ruminants moved during the period 2012–2020 were moved for slaughter purposes and thus abattoirs could act as important sentinels for livestock disease surveillance, including zoonotic diseases.

It is recommended that all animals permitted for transportation for slaughter purposes should neither be returned to farms nor sold to other farmers for breeding or fattening purposes, a situation that makes abattoirs dead-end destinations for disease transmission. However, in Uganda, the majority of the individuals involved in the business of animal transportation lack requisite grounding in principles of animal disease transmission risk reduction [2]. Consequently, from our observations in Uganda, additional bureaucratic steps, such as obtaining a letter of “No Objection” from the veterinary officials in the destination district make most livestock traders dishonestly seek the animal transportation permit for slaughter purposes even when the purpose is for breeding. In an attempt to minimize transportation costs, unscrupulous animal traders often obtain AMPs to move animals for slaughter even when some or all the animals transported are indeed destined for breeding purposes. Driven by their sole incentive to make money, most animal traders in Uganda will sell the animals destined for slaughter to the highest bidder, who is often the livestock breeder. Although widely undocumented, there has been a number of outbreaks of transboundary animal diseases, such as PPR and FMD, connected to the purchase of livestock from abattoirs by farmers, especially in districts near the big abattoirs, such as Wakiso district.

Likewise, the districts that have very high tendencies to send out animals to other districts are likely to spread infections in case they have infectious animals and could therefore be targeted for interventions, such as biosecurity measures and vaccination against priority diseases, to reduce the likelihood of transmission to other areas.

There were districts, such as, Kampala, Wakiso, Mukono, Kiruhura, Lira, and Kaberamaido with exceptionally high degree centrality and betweenness centrality. Such districts are very likely to act as spillways that enable the rapid flow of infectious disease agents to other districts that would have otherwise been poorly connected. Once such districts are carefully identified, they could be targeted with interventions to increase chances of disrupting the flow of potentially infectious animals and thus reduce the impact and extent of disease outbreaks [23], for instance quarantine facilities. Such districts that have very high hub score values are connected to majority of other influential districts in the country. Coupled with inadequate biosecurity measures at the slaughter houses or slaughter slabs in most towns in Uganda, environmental contamination with infectious disease pathogens is very likely and thus the identified hubs could serve as potential targets for sentinel surveillance [41].

The central region was the most favorite destination of animals largely because of the urban and peri-urban nature of the central region in Uganda which is associated with high demand for livestock and livestock products. This is similar to an observation made by a study on cattle movement networks in Uganda [6]. Most of the small ruminants were shipped for purposes of slaughter followed by breeding. As has been reported elsewhere, most small ruminants reared in Uganda are the indigenous type and therefore, the motivation for their rearing is for sale, social functions and family consumption to provide animal-source proteins. Moreover, small ruminant keepers in Uganda do not frequently restock animals as they often maintain and multiply their own stocks as previously reported [42].

The northern region of Uganda was identified as a general source of animals for slaughter in the rest of the country. Districts, such as Nabilatuk (Karamoja subregion), Lira, and Kaberamaido were among the most important districts in the dissemination of animals to other regions especially central and western regions. Lira and Kaberamaido districts have previously been identified as important districts in the cattle movement networks in Uganda [24]. Because of this, it is not surprising that the northern region in Uganda, especially the Karamoja subregion, has previously been blamed for being the source of small ruminant diseases, such as, PPR to other regions especially the central and southwestern regions [25]. Animal movements provide a golden opportunity to spread diseases over long distances in the shortest time possible especially if no biosecurity measures are available to minimize this risk as previously reported [14].

The small ruminant movement networks were more fragmented in the earlier years (2012–2016), however, the networks became strongly connected thereafter, an observation that is generally consistent with previous studies in Uganda [6, 24]. Despite no obvious temporal trends in the number of movement transactions over time, there were three distinct periods of high volume of small ruminants moved that coincided with the monthly shipment frequency (Figures 2 and 3). The observed peaks of high small ruminant movements coincide with months with festivities, for examples between March and April (Easter holiday) and around December (Christmas holiday). Hypothetically, if an infectious disease was introduced into one of the districts with the highest connectivity, the fastest spread of an outbreak would have been observed in this period 2017 and 2018. Indeed, this observation coincides with the shift in PPR focus from the Karamoja subregion to central and southwestern Uganda resulting in the first major outbreak reported in western Uganda in the same period [25]. The rapid increase in the frequency and number and of animals moved between regions in the periods 2018 and 2020 also coincided with the highest number of outbreaks of PPR reported in multiple districts in Uganda. The progressive increase in the number and frequency of small ruminants especially into urban districts follows the progressive increase in both human and livestock populations over time [9].

Interestingly, similar to what has been previously reported by Hasahya et al. [24], districts at international border points and those that make up cities and urban centres in Uganda were identified as the most important players in the small ruminant movement network. For example, districts, such as Amuru (border Uganda–South Sudan), Tororo (border Uganda–Kenya), Kasese (border Uganda–Democratic Republic of Congo), and Isingiro (border Uganda–Tanzania border) were among the districts that were influential in the small ruminant movement network. Urban centres and cities in Uganda have a relatively higher human population which in turn drives the demand for small ruminant meat to feed the urban dwellers. Moreover, there is a lot of human activity at international borders which facilitates trade of animals and some of the animals, maybe headed for export through illegal or legal means especially around the porous Ugandan borders [4]. For the case of transboundary animal diseases, such as PPR and FMD, the observed influence of some districts at the border points indicates a potential risk of spread of diseases from Uganda to other countries and vice versa. The trucks moving animals to markets and the traders themselves can facilitate dissemination of infectious diseases, such as PPR and FMD [4]. This observation of increased flow of small ruminants to districts along the international borders calls for more strict regulation of livestock movement by measures, such as establishing quarantine stations to minimize the potential likelihood of disease introduction into Uganda or vice-versa.

## 6. Conclusions and Recommendations

The districts that were identified as influential in the small ruminant networks can be good starting points to correctly institute animal disease control measures especially quarantine, vaccination, and enhanced biosecurity. Such influential districts in networks have previously been linked with the likelihood of driving the spread of infectious diseases in a very short time because of how quickly animals from them can potentially reach many districts in the country. The districts, such as Kampala, Wakiso, Lira, and Kaberamaido that demonstrated high levels of connectivity, especially by the different centrality measures should be prioritized for surveillance, control activities to increase the impact, and effectiveness of such activities. Districts with high degree centrality and betweenness would increase the accuracy and sensitivity of active surveillance efforts other than blindly implementing such activities. This would in turn improve timely detection of disease outbreaks and reduce the spatial extent and impact; improving the profitability of small ruminant production venture.

## Figures and Tables

**Table 1:** Some of the key measures of centrality used to describe network graphs.

Parameter	Description	Reference
General network terminologies		
Node	The unit of interest in the network analysis for example individual entities, farms, districts, etc.	[26]
Edge	Connection between two nodes in the network graph	[26]
Static network	Snapshot of a network that contains all nodes and edges for a given duration of observation time	[27]
Dynamic network	Captures the structural changes in both edges and nodes over time	[27]
Node-level metrics		
Degree	The total number of edges connected to a given node	[28]
In-degree	The total number of connections directed to a given node	[26]
Out-degree	The total number of connections directed away from a given node	[26]
Closeness centrality	This is a measure of how near a given node is to other nodes computed as the mean length of the shortest paths from one node to each other nodes	[28]
Betweenness	This quantifies the number of times a given node acts as a bridge between two other nodes along the shortest path	[29]
Eigenvector Centrality	Measures the importance of a particular node proportional to centrality scores of all its neighbors: a node is important if its neighbors are important	[29]
PageRank	PageRank's logic is comparable to that of eigenvector centrality, except it makes a significant distinction: the importance of a node's connection to an important source varies depending on how many or how few links that source has. If there are a lot of links, the source is penalized because its importance is a bit diluted	[28]
Hubs and authority	The Hub score is an efficient measure of the node's ability to send out links whereas the authority score is associated with the node's ability to receive links. Just as in the eigenvector centrality, the importance of a hub or authority is hugely dependent on the corresponding connection. According to inventor of the method: “*A node is an authority if it is linked to by hubs; it is a hub if it links to authorities.”*	[29, 30]
Network-level metrics		
Diameter	The largest geodesic distance in the network, i.e., the highest number of edges in the shortest path between two nodes	[26]
Assortativity	Tendency of nodes to connect more with nodes that share similar characteristics	[26]

**Table 2:** Total number of small ruminants moved across districts as summarized by “purpose*”* and “mode of transportation” for the entire study period (January 2012–January 2020).

Mode of transportation	Purpose and total number of small ruminants moved				
	Breeding (%)	Slaughter (%)	Dowry (%)	Research (%)	Total (%)
Truck	1475 (48.6)	6979 (81.3)	2 (40)	1 (100)	8457 (72.7)
Trekking	1272 (41.9)	1118 (13.0)	2 (40)	0 (0)	2392 (20.6)
Motorcycle	191 (6.3)	439 (5.1)	1 (20)	0 (0)	631 (5.4)
Bicycle	99 (3.3)	28 (0.3)	0 (0)	0 (0)	127 (1.1)
Boat	0 (0)	23 (0.3)	0 (0)	0 (0)	23 (0.2)
Ferry	0 (0)	1 (0.0)	0 (0)	0 (0)	1 (0.0)
Overall	3037 (26.1)	8588 (73.8)	5 (0.0)	1 (0.0)	11,631 (10)

## Data Availability

The data that support the findings of this study are available on request from the corresponding author. The data are not publicly available due to privacy or ethical restrictions.

## Supporting Information

Supporting Information Figure S1: Map of Uganda showing centrality measure visualization on static network for small ruminant movements. The size and color of the node is proportionally scaled to represent hub score centrality. The connections between districts are weighted based on the frequency of livestock movement between them. The map was generated in R version 4.4.2 using open-source shapefiles <https://www.ubos.org/data-portals-2/>.
