# Supplementary material for: Network Analysis of Small Ruminant Movements in Uganda: Implications for Control of Transboundary Animal Diseases
Source: Transbound Emerg Dis. 2025 May 14;2025:7474495. doi: 10.1155/tbed/7474495 (PMC12094867; doi:10.1155/tbed/7474495)
Supplement: Supporting Information — Figure S1: Map of Uganda showing centrality measure visualization on static network for small ruminant movements. The size and color of the node is proportionally scaled to represent hub score centrality. The connections between districts are weighted based on the frequency of livestock movement between them. The map was generated in R version 4.4.2 using open-source shapefiles <https://www.ubos.org/data-portals-2/>. [file 7474495.f1.pdf]

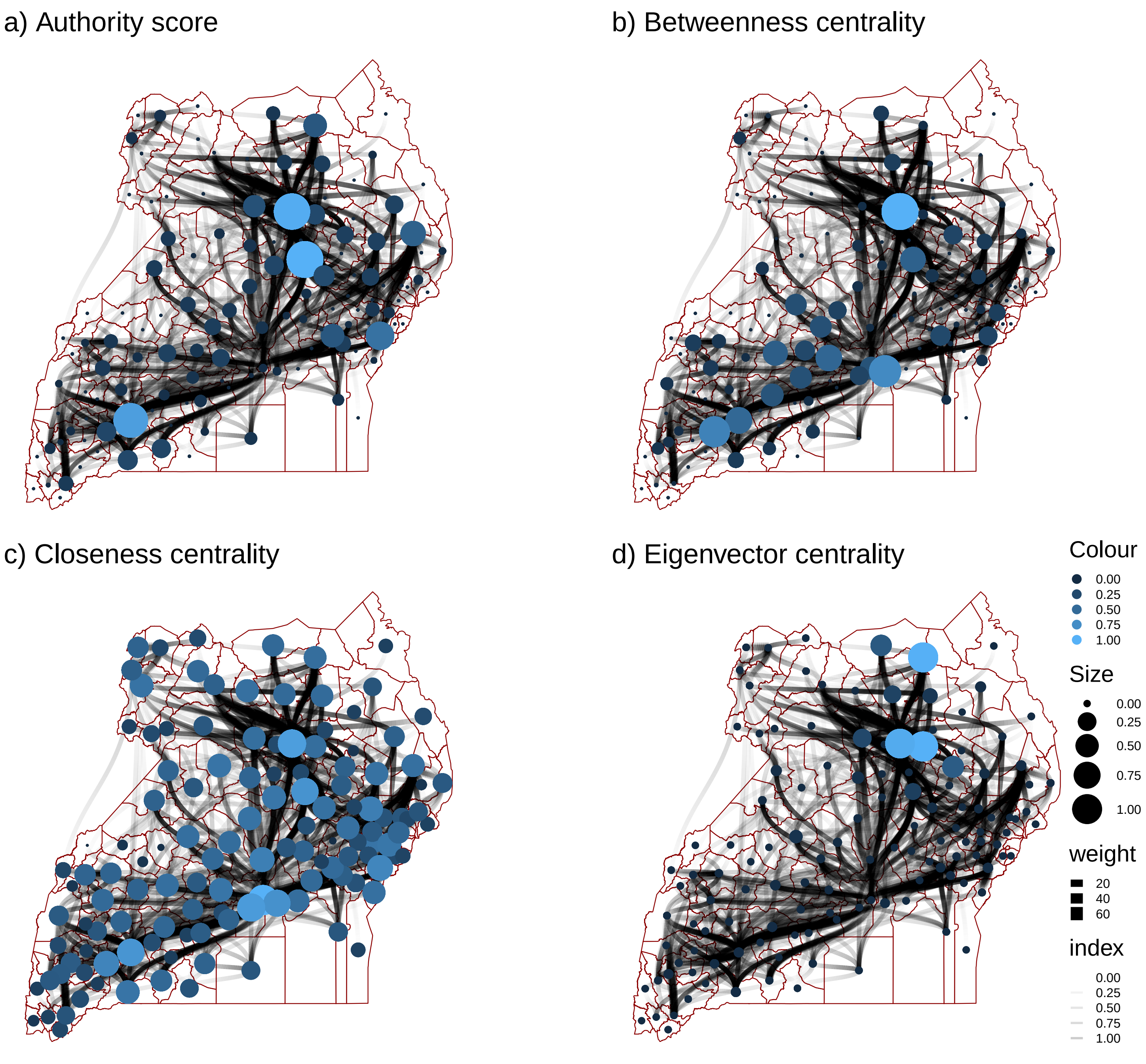
a) Authority score
b) Betweenness centrality
c) Closeness centrality
d) Eigenvector centrality
Colour
0.00
0.25
0.50
0.75
1.00
Size
0.00
0.25
0.50
0.75
1.00
weight
20
40
60
index
0.00
0.25
0.50
0.75
1.00
